# Psychological capital and alienation among patients with COVID-19 infection: the mediating role of social support

**DOI:** 10.1186/s12985-023-02055-6

**Published:** 2023-06-06

**Authors:** Chao Wu, Chun-yan He, Jia-ran Yan, Hong-li Zhang, Lu Li, Ci Tian, Nana Chen, Qing-yi Wang, Yu-hai Zhang, Hong-juan Lang

**Affiliations:** 1 Department of Nursing, Fourth Military Medical University, No.169 Changle West Road, Xi’an, 710032 Shaanxi China; 2 Department of Anesthesia Intensive Care Unit, The Second Affiliated Hospital, Fourth Military Medical University, Shaanxi, China; 3 Cardio-Thoracic Surgery, The 305Th Hospital of the Chinese People’s Liberation Army, Beijing, China; 4 Troops of the Chinese People’s Liberation Army, Sichuan, 32280 China; 5 Department of Foreign Languages, School of Basic Medicine, No.169 Changle West Road, Xi’an, 710032 Shaanxi China; 6 Department of Health Statistics, Fourth Military Medical University, No.169 Changle West Road, Xi’an, 710032 Shaanxi China

**Keywords:** Patients with COVID-19 infection, Psychological capital, Social support, Social alienation

## Abstract

**Background:**

COVID-19 infection continues all over the world, causing serious physical and psychological impacts to patients. Patients with COVID-19 infection suffer from various negative emotional experiences such as anxiety, depression, mania, and alienation, which seriously affect their normal life and is detrimental to the prognosis. Our study is aimed to investigate the effect of psychological capital on alienation among patients with COVID-19 and the mediating role of social support in this relationship.

**Methods:**

The data were collected in China by the convenient sampling. A sample of 259 COVID-19 patients completed the psychological capital, social support and social alienation scale and the structural equation model was adopted to verify the research hypotheses.

**Results:**

Psychological capital was significantly and negatively related to the COVID-19 patients’ social alienation (*p* < .01). And social support partially mediated the correlation between psychological capital and patients’ social alienation (*p* < .01).

**Conclusion:**

Psychological capital is critical to predicting COVID-19 patients’ social alienation. Social support plays an intermediary role and explains how psychological capital alleviates the sense of social alienation among patients with COVID-19 infection.

## Background

### Introduction

Currently, COVID-19 is still widespread around the world [4, 15] and the pandemic is still difficult to prevent and constrain in China as there are sporadic and clustered COVID-19 outbreaks taking place [1, 7]. Up to now, no effective drug for preventing COVID-19 is available [41]. The COVID-19 epidemic is a public health emergency that not only endangers patients’ physical health but also has an emotional impact on individuals and may result in related psychological problems [11, 22]. Patients with COVID-19 infection may experience common psychological symptoms, such as anxiety, insomnia, tension, as well as excessive attention to the body and disease recovery [39, 49]. The COVID-19 epidemic has created a situation where many factors that contribute to poor mental health are made worse. A study on *the Lancet* estimated an addition of 53.2 million cases of major depression and 76.2 million cases of anxiety disorder globally due to the COVID-19 pandemic [17]. These psychological problems are derived from the uncertainty about the prognosis of COVID-19 and the social discrimination against contagious novel coronavirus [36, 52]. These problems will result in a sense of alienation in patients. Social alienation has a negative impact on patients’ physical and mental health, and is detrimental to both the healing of sickness and the societal integration [8].

Social alienation is the automatic alienation that occurs between people and their families, other people, and society. This feeling of helplessness and loneliness leads to negative behaviors like avoidance and rejection [6, 33]. With COVID-19, the patients are labeled as something others fear of and become a symbol of awe and alienation for others, which has an adverse effect on patients’ physical and mental health and makes it difficult for them to reintegrate into society [38]. At present, there are few studies on social alienation of patients with COVID-19 infection, most of which focus on the negative emotion caused by COVID-19 [29, 45, 50]. Therefore, the purpose of our study is to carry out research to investigate the sense of alienation in patients with COVID-19 infection, in order to ascertain the severity of their social alienation and explore its underlying mechanisms, so as to provide references for alleviating the social alienation of COVID-19 patients and help them to maintain their mental health and to better integrate into society.

Psychological capital refers to the state of being psychologically positive as exhibited in individuals during their growth [3]. Good psychological capital can buffer the impact of negative emotions [9]. At present, psychological capital is a topic that has been extensively studied [25, 46, 58, 59]. Research shows that individuals with high psychological capital have better ability and resilience to withstand pressure in difficulties and adversity [35]. However, it’s less clear that whether psychological capital could ease social alienation among patients with COVID-19. Therefore, the primary objective of this study was to determine whether psychological capital could affect patients’ social alienation.

In addition, we attempted to identify the mechanisms through which psychological capital relieves COVID-19 patients’ alienation. Earlier studies have found that psychological capital is positively related to social support [18, 57]. This is because individuals with favorable psychological capital are more likely to seek external support when encountering difficulties [2]. Social alienation closes people off to the outside world by restricting their contact, and social support can help people feel less isolated from the outside world. When individuals receive social attention and support, they can feel more care and help from the outside, which helps to alleviate the social alienation [31]. Accordingly, we believe that psychological capital, social support and social alienation are interrelated, where social support may play an intermediary role between psychological capital and social alienation. Hence, the present study is designed to investigate the effect of psychological capital on patients’ feelings of social alienation and the mediating role of social support among patients with COVID-19. As such, we attempted to explore the impact of social alienation on COVID-19 patients and provide insights for reducing their social alienation.

### Literature review and hypotheses

#### Psychological capital and social alienation

Psychological capital is a psychological element and a psychological resource that could promote personal growth and development [51]. Psychological capital mainly includes four key dimensions: (a) Self-efficacy, a personal assessment of one’s ability to succeed. (b) Hope, which alludes to positive intentions or goals. (c) Tenacity, or a person’s unyielding spirit and perseverance. (d) Optimism, which is the state of being filled with confidence and optimism for the future [40].

Considering the infectious nature of the disease, COVID-19 patients need to be isolated in treatment after diagnosis [20]. Patients who are physically isolated develop a psychological distance to the outside world [14]. The society discriminates against the patients tested positive as carriers and communicators of the virus and this discrimination still exists even after their recovery, because patients have a possibility of reinfection. All of these make the patients feel socially excluded and less connected with others, and exhibit autistic emotions [23]. Social alienation mainly consists of four dominant dimensions: (a) Self-alienation, which is a denial of oneself. (b) Social isolation, the feeling of loneliness when interacting with others. (c) Powerlessness, the feeling brought on by a lack of accomplishment. (d) Meaningless, a sense of loss due to a lack of a goal or direction in life [60].

Research shows that good psychological capital can help individuals find solutions to problems [47]. Hope and optimism, in particular, can help individuals to eliminate the entrenched discrimination and respond to this discrimination with a positive attitude [26]. Here, we argue that psychological capital can alleviate social alienation. People who have supportive psychological capital can overcome their social isolation. Resilience can improve a person’s capacity to handle stress and withstand unfavorable feelings like powerlessness and helplessness in the face of challenges [16]. Therefore, based on these arguments, our first hypothesis is:

##### Hypothesis 1

Psychological capital is directly related to patients’ social alienation and can alleviate social alienation.

#### Social support as a mediator

Social support refers to the objective support from the outside to the individual and the subjective support perceived by the individual [53]. Previous researches show a close correlation between the psychological capital and social support [43, 53]. Individuals with high levels of psychological capital can seek more social support and help, which in turn promotes individual’s psychological capital [34].

A systematic review shows that social support was associated with positive mental and psychological health outcomes among health care workers during the COVID-19 pandemic [27]. And social support was identified as one of the protective factors against emotional loneliness during the COVID-19 pandemic [28]. The mental condition of COVID-19 patients with is closely linked to social support they received, and good social support can relieve their negative emotions such as tension and anxiety [21]. However, the studies currently do not have sufficient evidence in the impact of social support on patients’ social alienation. Thus, it is of great importance to investigate the relationship between social support and social alienation in COVID-19 patients.

Presently, there are limited investigations on the relations among psychological capital, social support and social alienation in patients with COVID-19. Therefore, it is crucial to investigate how social alienation affects COVID-19 patients in order to support their active social integration, preserve their mental health, and accelerate disease healing. We thus anticipate that social support functions as a mediator between psychological capital and social alienation. Individuals with strong psychological capital can seek greater social support, and good social support can in turn alleviate their social alienation. Taken together, we propose that:

##### Hypothesis 2

Social support mediates the relationship between psychological capital and social alienation.

## Methods

### Participants and data collection

Participants were collected by convenient sampling, and 284 COVID-19-infected patients from a shelter hospital were selected as study subjects. With the help of the head nurses, we distributed electronic questionnaires to patients in the shelter hospital from April to May 2022 in Shanghai. All the patients diagnosed with COVID-19 meet the inclusion criteria with those who refused to participate or were absent during the survey excluded. Participants’ informed consent was acquired before the survey was conducted. The time required to complete the phone-sent questionnaire was under control in just over 30 min. The questionnaire’s cover page reaffirmed the study’s objectives and importance. The survey was completed in an anonymous manner with a promise to protect the privacy and to use the data for research purpose only. Patients were informed that they could withdraw from the study at any time for any reason. Thirteen patients dropped out of the study midway through, seven questionnaires were determined to be incomplete, and five surveys were deemed invalid because of their excessive uniformity. After the questionnaires were collected, 259 of them were determined to be valid with an effective recovery rate of 91.97%.

### Measures

#### Psychological capital

Psychological capital was measured using the Psychological Capital Questionnaire (PCQ) [13]. PCQ is a higher-order construct consisting of 4 subscales, each comprised of 6 items (with a total of 24 items). The subscales include self-efficacy, hope, tenacity, and optimism. Example items include ‘I believe there are many different ways to solve the problem’ and ‘I believe I can analyze long-term problems and find solutions’. A 6-point Likert scale was adopted, ranging from 6 (totally agree) to 1 (totally disagree) with higher scores indicating higher psychological capital. The scale is widely used in Chinese population research and has good applicability [10, 54]. In our research, the scale also shows good reliability and validity; its Cronbach’s alpha coefficient was 0.87 and varied between 0.81 and 0.90 for each of the scale’s 4 dimensions.

#### Social support

Social support was measured using the Social Support Rate Scale (SSRS) [30]. SSRS is a higher-order construct consisting of 3 subscales, with a total of 10 questions items. The subscales fall into objective support (3 items), subjective support (4 items) and utilization of support (3 items). Example items include ’How many close friends do you have?’ and ‘Who comforted and cared for you when you were in trouble?’; Higher scores indicate higher social support. The scale is widely applied in Chinese populations, showing a good applicability [44, 58, 59]. The scale also has strong reliability and validity in our research with the Cronbach's alpha coefficient of 0.84, ranging between 0.83 and 0.91 across the 3 dimensions.

#### Social alienation

Social alienation was measured using the Social Alienation Scale (SAS) [33]. SAS is consisted of 4 subscales, totaling 15 items. The subscales include self-alienation (3 items), social isolation (5 items), powerlessness (4 items) and meaningless (3 items). Example items include ‘Few people really care about my feelings.’ and ‘I often feel lonely when I am with others.’ Higher scores indicate higher social alienation. The scale has good applicability among Chinese populations [60]. In our study, the scale also showed strong validity and reliability; its Cronbach's alpha coefficient was 0.93 and ranged between 0.82 and 0.91 for each of the scale's 4dimensions.

### Statistical analysis

IBM SPSS 26.0 was used by us to evaluate the data. Descriptive statistics were used to describe every variable. Pearson's correlation analysis was used to investigate the correlation among psychological capital, social support and social alienation. A two-step procedure of structural equation model was adopted to analyze the mediating effect of social support between psychological capital and social alienation using Mplus 8.3. To test our hypothesis, the measurement model and structural model were applied in two successive steps. We ran 2000 bootstrapping resamples adopting 95% confidence intervals (CI) to test the direct and indirect effect. All statistical analysis was two-side, and *p* < 0.05 was established as statistically significant. Several model fit indices were adopted to assess the adequacy of model fit: the chi-square test (χ2), the Tucker-Lewis index (TLI), the comparative fit index (CFI), the standardized root mean square residual (SRMR) and the root mean square error of approximation (RMSEA).

## Results

### Demographic characteristics of patients with COVID-19 infection and variables score

With the assistance of head nurses in the shelter hospital, 259 out of 284 patients completed the survey with a response rate of 91.97%, among whom 144 were male (55.60%) and 115 were female (44.40%). The subjects had an average age of 41.87 years (*SD* = 12.62); 97 subjects were from urban areas (37.45%) and 162 were from rural areas (62.55%); 184 patients had high-school degrees or less (71.04%), 73 had bachelor's degrees (28.19%), and 2 had master's degrees (0.77%); 10 were unmarried (3.86%), 236 were married (91.12%), and 13 were divorced or widowed (5.02%).

Table 1 shows the scores of the 3 scales and subscales. The psychological capital score of 259 patients with COVID-19 was (104.01 ± 14.89) with the social support score of (37.20 ± 7.88), and the social alienation score of (35.39 ± 4.64). The scores of other dimensions were presented in Table 1.

**Table 1:** Descriptive statistics and internal consistency of the scales

	Mean	*SD*	Min	Max	Cronbach’s α	Items
Psychological capital	104.01	14.89	43	144	0.87	24
Self-efficacy	27.45	4.25	14	36	0.81	6
Hope	26.10	4.98	12	36	0.90	6
Tenacity	24.62	4.35	7	36	0.81	6
Optimism	25.84	3.58	7	36	0.82	6
Social support	37.20	7.88	13	53	0.84	10
Objective support	18.59	4.01	6	24	0.91	3
Subjective support	9.30	3.21	6	30	0.83	4
Utilization of support	9.32	2.04	3	12	0.88	3
Social alienation	35.39	4.64	21	56	0.93	15
Self-alienation	6.68	1.47	3	12	0.91	3
Social isolation	11.03	2.21	5	19	0.84	5
Powerlessness	8.95	1.79	4	15	0.82	4
Meaningless	8.74	1.19	6	12	0.86	3

### Correlation analysis of psychological capital, social support and social alienation

Table 2 displays the Pearson correlation coefficients among variables in the survey. The results showed that psychological capital and its 4 dimensions have significantly positive correlations with social support (*r* = 0.58, *p* < 0.01; *r* = 0.54, *p* < 0.01;* r* = 0.58, *p* < 0.01;* r* = 0.50, *p* < 0.01;* r* = 0.36, *p* < 0.01) and have significantly negative correlations with social alienation (*r* = − 0.36, *p* < 0.01; *r* = − 0.34, *p* < 0.01;* r* = − 0.39, *p* < 0.01;* r* = − 0.38, *p* < 0.01;* r* = − 0.36, *p* < 0.01). Social alienation and its 4 dimensions had a negative correlation with social support (*r* = − 0.30, *p* < 0.01; *r* = − 0.31, *p* < 0.01;* r* = − 0.33, *p* < 0.01;* r* = − 0.34, *p* < 0.01).

**Table 2:** Correlations among the variables

		1	2	3	4	5	6	7	8	9	10	11	12	13
1	Psychological capital													
2	Self-efficacy	0.87^**^												
3	Hope	0.92^**^	0.81^**^											
4	Tenacity	0.89^**^	0.66^**^	0.75^**^										
5	Optimism	0.76^**^	0.54^**^	0.56^**^	0.70^**^									
6	Social support	0.58^**^	0.54^**^	0.58^**^	0.50^**^	0.36^**^								
7	Objective support	0.50^**^	0.46^**^	0.49^**^	0.44^**^	0.33^**^	0.89^**^							
8	Subjective support	0.39^**^	0.47^**^	0.41^**^	0.34^**^	0.21^**^	0.81^**^	0.66^**^						
9	Utilization of support	0.62^**^	0.54^**^	0.61^**^	0.54^**^	0.47^**^	0.84^**^	0.70^**^	0.65^**^					
10	Social alienation	− 0.36^**^	− 0.34^**^	− 0.39^**^	− 0.38^**^	− 0.36^**^	− 0.42^**^	− 0.37^**^	− 0.36^**^	− 0.39^**^				
11	Self-alienation	− 0.23^**^	− 0.34^**^	− 0.33^**^	− 0.34^**^	− 0.28^**^	− 0.30^**^	− 0.31^**^	− 0.33^**^	− 0.34^**^	0.84^**^			
12	Social isolation	− 0.25^**^	− 0.31^**^	− 0.35^**^	− 0.27^**^	− 0.31^**^	− 0.28^**^	− 0.28^**^	− 0.32^**^	− 0.38^**^	0.74^**^	0.76^**^		
13	Powerlessness	− 0.22^**^	− 0.24^**^	− 0.30^**^	− 0.27^**^	− 0.24^**^	− 0.32^**^	− 0.34^**^	− 0.33^**^	− 0.25^**^	0.88^**^	0.70^**^	0.76^**^	
14	Meaningless	− 0.36^**^	− 0.32^**^	− 0.35^**^	− 0.35^**^	− 0.41^**^	− 0.42^**^	− 0.34^**^	− 0.32^**^	− 0.47^**^	0.76^**^	0.73^**^	0.75^**^	0.80^**^

### Verification of research hypotheses

**Fig. 1:**
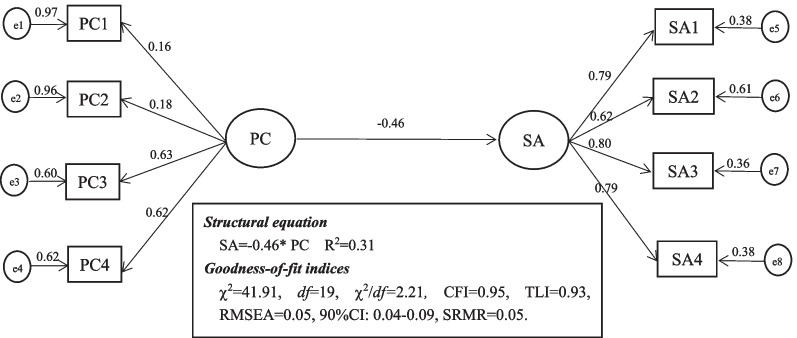
Direct effect model. PC, Psychological capital; e1-e4, manifest variables of the four dimensions of psychological capital; SA, social alienation; e5-e8, manifest variables of the four dimensions of social alienation

To verify hypothesis 1, we firstly tested the direct effect model, with the results indicating a good fit in the direct effect model: χ^2^ = 41.91, *df* = 19, χ^2^/*df* = 2.21*,* CFI = 0.95, TLI = 0.93, RMSEA = 0.05, 90%CI: 0.04–0.09, SRMR = 0.05 (*p* < 0.01) (as shown in Fig. 1). Psychological capital was negatively correlated with social alienation (*β* = − 0.46, *p* < 0.01). Then we performed 2000 bootstrapping resamples to justify the 95% CI of the total direct effect of psychological capital on social alienation, which showed 95% CI for the total direct effect (− 0.65, − 0.27). And psychological capital could justify the 31% variance of social alienation.

**Fig. 2:**
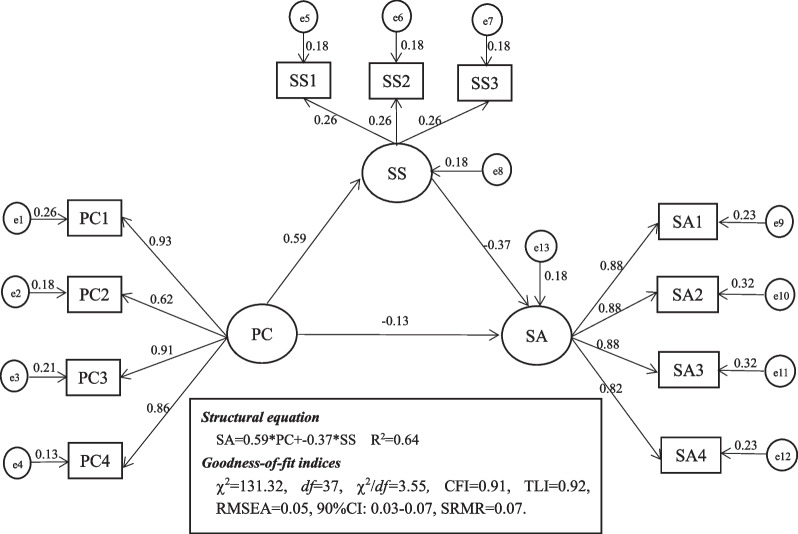
Mediation Model. PC, Psychological Capital; SS, Social Support; SA, Social Alienation

Next, we tested a mediating effect model to verify hypothesis 2 that whether social support mediates psychological capital and social alienation. We repeated this process 2000 times to obtain an empirical approximation of the sampling distribution, and to obtain estimates and confidence intervals for this indirect effect. The confirmatory factor analysis revealed that the three-factor model had an adequate fit to the data: χ^2^ = 131.32, *df* = 37, χ^2^/ *df* = 3.55*,* CFI = 0.91, TLI = 0.92, RMSEA = 0.05, 90%CI: 0.03–0.07, SRMR = 0.07 (*p* < 0.01) (as shown in Fig. 2). Psychological capital was positively related to social support (*β* = 0.59, *p* < 0.01), while negatively related with social alienation (*β* = − 0.13, *p* < 0.01). And social support also showed a negative correlation with social alienation (*β* = − 0.37, *p* < 0.01). We performed 2000 bootstrapping resamples to justify the 95% CI of the indirect effect of psychological capital on social alienation via social support. The results showed that the 95% CI of the mediating effect was (− 0.27, − 0.17), with the total effect of − 0.46 and the indirect effect of − 0.22. The indirect effect accounted for 47.82% of the total effect of psychological capital on social alienation (Table 3).

**Table 3:** Confidence interval of mediating effect value in the mediated model (2000 bootstrap samples)

Model path	Estimate	95%CI	
		LLCI	ULCI
PC → SA	− 0.46	− 0.65	− 0.27
PC → SS → SA	− 0.22	− 0.27	− 0.17

## Discussion

### The direct effect of psychological capital on social alienation

Through the investigation of 259 COVID-19 patients, we found that their psychological capital can directly predict social alienation. Psychological capital is a positive psychological state which can buffer the negative effects of negative emotions [42]. The negative correlation between psychological capital and social alienation was verified by the correlation analysis in patients with COVID-19 infection (*p* < 0.01). Following an infection, the patient is very contagious, and the effects of this COVID-19 remained unclear at this moment [19, 61]. Additionally, being isolated in such an unfamiliar environment as the shelter hospital, it is easy for the patients to develop negative psychological conditions such as closure, refusal to communicate with others, fear and so on [12]. If with positive psychological capital, when facing difficulties, these patients would show positive responses such as hope, optimism, resilience, and self-efficacy. These positive reactions will encourage them to accept the reality of infection, actively receive treatment against the disease, and avoid the alienation feelings [48]. Specifically, when the patient is optimistic about his or her treatment and prognosis rather than abandons himself, it helps to create a sense of self-worth and avoid being isolated from the external environment.

Research shows that mindfulness meditation might be a viable low-cost intervention to mitigate the psychological impact of the COVID-19 crisis [62, 63]. Therefore, COVID-19 patients should actively raise their psychological capital through practices like the stress reduction and mindfulness meditation [24, 62, 63]. This will help them recover quickly from adversity, failure, conflict and pressure, and actively face various problems and properly solve them. Medical professionals should assist COVID-19 patients to improve their psychological capital, taking into account issues such as their health problems, interpersonal relationships and economic difficulties, and eventually help them to actively integrate into the society.

### The intermediary effect of social support between psychological capital and social alienation

Further investigation revealed that social support was a mediator factor between psychological capital and social alienation in patients with COVID-19 infection, accounting for 47.82% of the variance in our study. Patients with good psychological capital can actively seek out support from others and can get access to more social support resources. Thus, our findings are in consistence with that of [31, 32], and that of Yarcheski & Mahon [55] suggesting that the higher the level of perceived social support, the higher the level of psychological capital.

Calati et al. [5] proposed that social support can negatively predict people's sense of alienation, which means that the more the social support, the less lonely people tend to feel. Social support can alleviate the negative emotional experience of patients with COVID-19; specifically, the more external support they receive from friends, coworkers, and family members, the less social alienation they feel [56].

Lack of adequate psychological capital among patients will result in a loss in interpersonal and adaptive abilities after the disease. Subsequently, it will make people feel isolated from others and reluctant to seek social help; and ultimately increase their sense of social alienation. On the contrary, a patient who has a strong psychological capital can adjust to changes well, actively fight against the disease, and seek help from the outside. This improved social capital enables people to obtain social support, foster a sense of belonging, and feel less isolated, thus leading to the lowered sense of social alienation.

Therefore, medical personnel should be completely aware of the critical role that social support plays in reducing social alienation in COVID-19 patients, as the patients are vulnerable to unfair treatment such as social discrimination, exclusion, isolation and alienation [62, 63]. Medical personnel should provide support especially psychological assistance to patients. They could allay the patients' worries by reassuring them and sharing successful cases of patients recovering from COVID-19.

## Conclusions

Psychological capital is critical to predicting COVID-19 patients' social alienation. Social support plays an intermediary role and explains how psychological capital alleviates the sense of social alienation among patients with COVID-19 infection.

## Data Availability

The datasets generated and analyzed during the current study are not publicly available due to the protection of the privacy of consulting experts but are available from the corresponding author (906963251@qq.com) on reasonable request.
